# Prevalence of hepatitis B and C viral co-infections among HIV-1 infected individuals in Nairobi, Kenya

**DOI:** 10.1186/1756-0500-6-363

**Published:** 2013-09-09

**Authors:** Beatrice Mukami Muriuki, Michael Muita Gicheru, Dorcas Wachira, Anthony Kebira Nyamache, Samoel Ashimosi Khamadi

**Affiliations:** 1 Department of Zoological Sciences, Kenyatta University, Nairobi, Kenya; 2 Department of Plant and Microbial sciences, Kenyatta University, Nairobi, Kenya; 3 Kenya Medical Research Institute, Centre for Virus Research, Nairobi, Kenya

**Keywords:** Hepatitis, HIV-1, ELISA

## Abstract

**Background:**

Hepatitis B virus (HBV) and Hepatitis C virus (HCV) co-infections among HIV-1 infected individuals are growing worldwide health problems characterized by lack of effective vaccines, need for expensive treatment, chronicity of morbidity and associated mortality. Their prevalence and distribution patterns continue to vary across geographical locations with high prevalence being detected among high risk populations. To determine the prevalence of HBV and HCV among HIV-1 infected individuals, blood samples were collected from consenting study subjects visiting comprehensive HIV clinics in Nairobi during the period between October and December 2009.

**Methods:**

Blood samples from volunteers were screened with ELISA tests for detecting HIV, HBV surface antigen (HBsAg) and anti-HCV antibodies.

**Results:**

In a total of three (300) hundred infected individuals consisting of 129 (43%) males and 171 (57%) females 15.3% (46/300) were HIV-1 co-infected with either HBV or HCV or both, 10.3% (31/300) with HIV-1 and HCV and 6% (18/300) with HIV-1 and HBV infections. However, only three individuals (1%) were coinfected with the three viruses (HIV/HBV/HCV).

**Conclusion:**

Though, low levels of co-infection with all three viruses were reported, there could be higher prevalence rates than reported here especially among high risk populations.

## Background

Human immunodeficiency Virus (HIV), Hepatitis B virus (HBV), and Hepatitis C virus (HCV), are the three most common chronic viral infections all over the world. They share similar transmission routes including sexual, blood-blood contact, and injecting drug usage [1,2]. Co-infection with HIV and HCV and/or HBV is very common in certain populations, such as intravenous drug users (IDUs) who often share the contaminated needles/syringes for intravenous drug injection. It has been reported that the world prevalence of HIV-HCV co-infection among IDUs can surpass 90% in certain populations [3,4]. Besides, the rates of HIV-HBV co-infection are reported as high as 10–20% in countries where HBV infection is either endemic or intermediate to high HBV cases [5]. It has been observed that HBV/HIV co infection leads to increased morbidity and mortality as compared to HIV or HBV mono-infections [5]. The ever increasing burden of these infections has become a growing concern [6]. With increased access to antiretroviral drugs for HIV patients, migrating populations and social networking by intravenous drug use, cases of HBV and HCV co infections have been on the rise [7], coupled with the dramatic rise in survival rates of these individuals [8]. As a result of these factors, cases of hepatic diseases have also been on the rise [9]. Studies show that HIV co infection adversely impacts on the natural history of HBV and HCV [7] by accelerating progression to chronic live disease due to drug-related hepatotoxicity and hepatitis reactivation [10-12]. At this stage, most patients are likely to die due to liver-related diseases compared to those without HIV infection [13].

In Kenya, the HIV-1 epidemic has been well documented. However, little data exists on HBV and HCV co infection among HIV-1 positive patients [14]. This study was carried out among HIV positive patients who were accessing CD4 testing services, to determine the levels of HBV and HCV co infections. This was done with the knowledge that such patients are at a high risk of a rapid disease progression coupled with development of liver cirrhosis and hepatocellular carcinoma [15].

## Methods

Between October and December 2009, a cross sectional study was carried out involving three (300) hundred consenting HIV-1 infected patients. The volunteers were recruited after providing written consent i.e. for persons aged above 18 years and assent from guardians and mothers of persons aged below 18 years. The enrolled volunteers were aged between 4–59 years of age. The participants were recruited through stratified sampling from nine Nairobi-based health centres namely; Baraka, Ngong, Pumwani, Ongata Rongai, Kangemi, Huruma, Kamiti, Ngaira and Waithaka. Blood was collected aseptically into 10 ml vacutainer tubes (Becton Dickson, New Jersey USA) for biochemical, CD4+ count and viral serology tests. The CD4+ T lymphocytes assay was performed within three hours of collection, while serum for serological assays for hepatitis C and B markers was stored at −20°C until the time for assay [16]. CD4+T lymphocytes count was determined by flow cytometry using Becton Dickson Facs calibur machine [17]. The determination of anti hepatitis C virus IgG antibody (anti HCV) and Hepatitis B antigen (HBsAg) were conducted by use of antibody capture ELISA, enzyme-linked immunosorbent assay kit (Hepanostika HBsAg) (Murex Biotech Ltd, Dartford, UK) [18] and Murex anti-HCV kit for Hepatitis C virus, (Murex anti-HCV version 4.0) [19]. HIV-1 and HIV-2 Vironostika HIV Uni-Form II Ag/Ab (Bio-Merieux, Boxtel, The Netherlands) following the manufacturer’s instructions.

### Ethical considerations

This study was ethically approved by Kenya Medical Research Institute (KEMRI) National Scientific Steering Committee SCC reference no. 1394 before it was executed.

### Data analysis

All generated data was entered into a database, cleaned and analyzed using SPSS version 11.5. The sero-prevalence for HBV and HCV were expressed as a percentage for the entire study group. Pearsons Chi square was used to test the association between level of immunosuppression and HBV/HCV infection. Simple linear correlation analysis was used to determine the association between age and HBV/HCV infection.

## Results

Three (300) hundred HIV patients comprising 129 (43%) males and 171 (57%) females were enrolled in this study. Their age ranged between 4–59 years with a mean age of 33.92 (±8.96) years with men 36.0 (±8.7) and 32.6 (±8.97) years for females (Table 1). There were 48 (16%) co-infections with 18 (56%) HIV/HBV, 30 (10.%) HIV/HCV, with 3 (1%) of those infected being detected with HIV/HBV/HCV coinfections. However, 248 (82.7%) were HIV monoinfected (see Table 1). In relation to gender, there was no difference in gender in dual infections of either HBV/HIV or HCV/HIV. However, males had the highest prevalence rates of HBV infection (55%) with females leading in HCV infections (53.3%). Among those infected, majority of them were aged 25 and 40 years (see Table 2). In addition, the mean CD4 counts in HBV/HIV co-infected patients were low compared to those either HCV/HIV co-infected patients or among HIV mono-infections (see Table 1).

**Table 1:** Characteristics of HIV positive, HIV/HCV and HIV/HBV co-infected individuals

**Categories**	**All n=(300)**	**Male**	**Female**	**HIV-1 only**	**HIV-1/HBV**	**HIV-1/HCV**	**HIV-1/HBV/HCV**	**p=value**
Age(yrs)[mean (S.D)]	33.9 (9.1)	35.7 (8.7)	32.6 (9)	34.24 (9.138)	34.8 (6.5)	30.6 (8.3)	33.3 (7.2)	0.001
CD4+ T cell count (Cells/mm3) (Mean)	388 (±262.95)	375 (±250.2)	398 (±226.9)	400.7 (±270.1)	252.2	325.8 (±228.7)	576.7 (±213.5)	0.02
HBV (%)		10/18 (55%)	8/18 (45%)					
HCV (%)		14/30 (46.7%)	16/30 (53.3%)					
Patients [n] (%)	300 (100)	129 (43.0)	171 (57)	248 (82.7)	18 (6)	30 (10%)	3 (1)	

**Table 2:** Prevalence of HIV-1, HBV, HCV coinfections by age and CD4+ counts

**Age group**	**(n/300)**	**HIV**	**HBV/HCV**	**HBV**	**HCV**	**Average CD4 count**
1-5	2	2	-	-	-	1211
6-10	2	1	-	-	1	370
11-15	2	2	-	-	-	537
16-20	4	3	-	-	1	409
21-25	40	31	1	2	6	461
26-30	73	61	-	4	8 (11.1)	401
31-35	61	52	-	3	6	350
36-40	48	35	2	5	6 (12.8)	356
41-45	40	34	-	4	2	380
46-50	18	17	-	-	1	272
51-55	10	10	-	-	-	432
56-60	3	3	-	-	-	352
**Total**	**300**	**248**	**3**	**18**	**31**	

## Discussion

With the increased access to antiretroviral therapy in resource limited settings, people living with HIV/AIDS will continue to live longer. However, morbidity and mortality due to co-infections with other viruses will increasingly become important. Although co infections with HBV and HCV among HIV positive patients is well documented in developing countries, the demographics and impact of these infections are not well defined in low resource countries like Kenya. The need for new data on hepatitis coinfections to guide health policy is on management of HIV coinfected a patient is paramount [19].

In this study, we examined the prevalence of HBV and HCV among HIV infected patients seeking CD4 testing services. From the results, it is clear that the co infection rates of HIV-infected patients with HBV and HCV was high (>6%). This number is high even though the study participants were persons who were seeking CD4 testing services i.e. probably immunosuppressed and not representative of the general population. This has an indication that hepatitis infection in HIV-infected persons may be higher than that of the general population. Co –infection with HCV was also high (10%). This was higher than what we could expect in the general population i.e. about 5% [20,21]. Compared to previous studies in Kenya on similar populations, these findings were contrarily higher 1.1% [22]; 0.7% [23], including those from Zambia (2.2%) [19], Cote d’voire (1.2%) [24], Gambia (0.6%) [25], Malawi (5.7%) [26,27], Nigeria (4.8%) [28], Rwanda (4.9%) [29], Senegal (1.6%) [30] Uganda (0.6%) [31] and Zimbabwe (0.8%) [31] (Table 3). The higher rates could probably due to increased free access to antiretroviral therapy and diverse study subjects. However, the high estimate of HCV prevalence among HIV infected patients in this study were similar to those obtained in Tanzania; [21,32] but less to those obtained in Argentina [33].

**Table 3:** Surveys of hepatitis B virus surface antigen (HBsAg) and hepatitis C antibody (HCV Ab) prevalence in HIV-infected persons conducted in Kenya and neighboring sub-Saharan Africa countries, 1996-2013

					**Prevalence rates**		
**Author**	**Publication year**	**Country**	**Population**	**Sample size**	**HBsAg (%)**	**HCV (%)**	**HCV/HBV/HIV (%)**
Muriuki et al. (current study)	2013	Kenya	Outpatient	300	6	10.3	
Mutuma et al. [34]	2011	Kenya	Outpatient	51	8.8		
Kapembwa et al. [19]	2011	Zambia	Inpatient	323	9.9	2.2	
Moore et al. [26]	2010	Malawi	Inpatient	300	6.7	5.7	
Di Bisceglie et al. [35]	2010	South Africa	Outpatient	502	4.8		
Mboto et al. [25]	2010	The Gambia	Outpatient	1500		0.6	
Kerubo et al. [23]	2010	Kenya	outpatients			0.7	0.3
Belay et al. [36]	2010	Ethiopia	Blood donors	6321	4.7	0.7	
Lukhwareni et al. [37]	2009	South Africa	Outpatient	192	22.9		
Oshitani et al. [38]	1996	Zambia	Outpatient	340	7.1		
Harania et al. [22]	2008	Kenya	Outpatient	378	6.1	1.1	
Nyirenda et al. [27]	2008	Malawi	Inpatient	226	17.5	4.5	
Otegbayo et al. [28]	2008	Nigeria	Outpatient	1779	11.9	4.8	
Diop-Niaye et al. [30]	2008	Senegal	Outpatient	363	16.8	1.6	
Shimeli et al. [39]	2008	Ethiopia	Outpatient	305	3.9		
Fimhaber et al. [35]	2008	South Africa	Outpatient	502	4.8		
Hoffmann et al. [40]	2008	South Africa	Outpatient	537	19.7		
Nagu et al. [20]	2008	Tanzania	Outpatient	260	17.3	18.1	
Pirillo et al. [29]	2007	Uganda	Antenatal	164	4.9	0.6	
Telatela et al. [32]	2007	Tanzania	Outpatient		15	13.8	
Lesi et al. [41]	2007	Nigeria	outpatients	240	9.2	5.8	1.5
Pirillo et al. [29]	2007	Rwanda	antenatal	82	2.4	4.9	
Wester et al. [42]	2006	Botswana	Outpatient	160	10.6		
Okoth et al. [43]	2006	Kenya	Outpatient		15		
Otedo et al. [44]	2004	Kenya	Outpatient	599	47		53
Ejele et al. [45]	2004	Nigeria	outpatient	342	9.7		1
Rouet et al. [24]	2004	Cote d’Ivoire	Antenatal	501	9	1.2	
Mustapha et al. [46]	2004	Gombe Nigeria	outpatients	200	26.5		
Kallestrup et al. [31]	2003	Zimbabwe	Outpatient	124		0.8	
Kasolo et al. [47]	2003	Zambia	Antenatal	214	31.3		
Ampofo et al. [42]	2002	Nigeria	blood donors		15	8.4	
Sud et al. [48]	2001	Nigeria	outpatients	80	22.2		
Lodenyo et al. [44]	2000	South Africa	Outpatient	100	6	1	
Rahlenbeck et al. [49]	1997	Ethiopia	outpatients	2186	14.4		
Oshitani et al. [38]	1996	Zambia	Outpatient	340	7.1		
Fainboim et al. [33]	1999	Argentina	outpatients	484	14.5	58.5	

The HIV/HBV co infection rates (6%) detected in this study was found to be consistent with findings from other studies carried out in Kenya [22,50], Zambia [19,34], Cote d’ Ivore [24], Malawi [25], Nigeria [38], Ethiopia [41] and South Africa [39]. However, these findings were low compared to those previous obtained in Kenya (55.8%) among liver failure patients [43,44] Tanzania [32,51], Zambia [47], Botswana [42], Malawi [27], Nigeria [28,42,46,48], Ethiopia [40], Argentina [34] South Africa [37,40] (Table 3). These prevalence rates were also found to be higher than those obtained in Ethiopia [35,52], South Africa [35,53], Uganda and Rwanda [29]. The observed diverse prevalence rates across different countries were associated with the diversity of patients from different population groups, sample size, test kit sensitivity and specificity [52].

The HIV co-infection rates as per gender in this study was found not to be significantly higher among male study subjects (19.2%) as compared to their female counterparts (14.3%) (p>0.061). This finding was comparable to reports from elsewhere [22]. This observation may have been accounted for by the fact that men are more likely to have multiple sex partners and also practice unprotected sex due to the polygamous nature of their relationships.

We also observed a significant high prevalence in HCV antibody among HIV infected patients. The probable reason could be due to the shared modes of transmission of both viruses in the study patients. The prevalence of HIV/HCV was also found to be higher in Males (11.6%) as compared to females (9.4%). However, the difference was not significant (p>0.055). This observation was similar to previous studies conducted in Kenya [23], Nigeria [4,21] and contrarily to other findings of older females being more co- infected [23,51].

Three patients (1%) were found co-infected with trio HIV, HBV and HCV infections in this study. These findings were similar to those obtained previously in Kenya [23,25], Nigeria [53] and Ethiopia [29] indicating a maintained low rates of these trio infections [22,23,25,35,36] (Table 3).

## Conclusions

In spite of carrying out the study only among HIV-infected patients, it was evident that co-infection rates with HIV and HCV and/or HBV are higher among HIV infected individuals than populations that are HIV negative. There is therefore a need for constant surveillance of these infections that pose a challenge in vaccine design and treatment options.

## Abbreviations

HBV: Hepatitis B virus; HCV: Hepatitis C virus; HBsAg: Hepatitis B surface antigen; Anti-HCV: Anti-HCV antibody; ELISA: Enzyme-linked immunosorbent assay.

## Competing interests

The authors declare that they have no competing interests.

## Authors’ contributions

BMM participated in data collection and carried out the analysis together with AKN, NW, NMG and SAK. They all participated in compiling the manuscript. ANK helped in preparing Tables. SAK and AKN assisted with the overall study design and supervised the statistical analysis. NMG and AKN were responsible for the overall supervision of the study and together with SAK supervised laboratory work. All authors read and approved the final manuscript.
